# Supplementary material for: The Succinate Receptor SUCNR1 Resides at the Endoplasmic Reticulum and Relocates to the Plasma Membrane in Hypoxic Conditions
Source: Cells. 2022 Jul 13;11(14):2185. doi: 10.3390/cells11142185 (PMC9321536; doi:10.3390/cells11142185)
Supplement: Supplementary file 1 [file cells-11-02185-s001.zip › cells-1751688-supplementary.pdf]

The succinate receptor SUCNR1 resides at the endoplasmic reticulum and relocates to the plasma membrane in hypoxic conditions

Melanie Sanchez[1,†], David Hamel[2,†], Emmanuel Bajon[3,†], François Duhamel[2], Vikrant K. Bhosle[1,4], Tang Zhu[3], Jose Carlos Rivera[5], Rabah Dabouz[1], Mathieu Nadeau-Vallée[2], Nicholas Sitaras[2], David-Étienne Tremblay[2], Samy Omri[5], Tiffany Habelrih[2], Raphael Rouget[1], Xin Hou[3], Fernand Gobeil[6], Jean-Sebastien Joyal[3,5], Przemyslaw Sapieha[5], Grant Mitchell[3], Alfredo Ribeiro-Da-Silva[1], Mohammad Ali Mohammad Nezhady[3,7,8,*] and Sylvain Chemtob[1,2,3,5,8,*]

# - SUPPLEMENTARY MATERIAL -

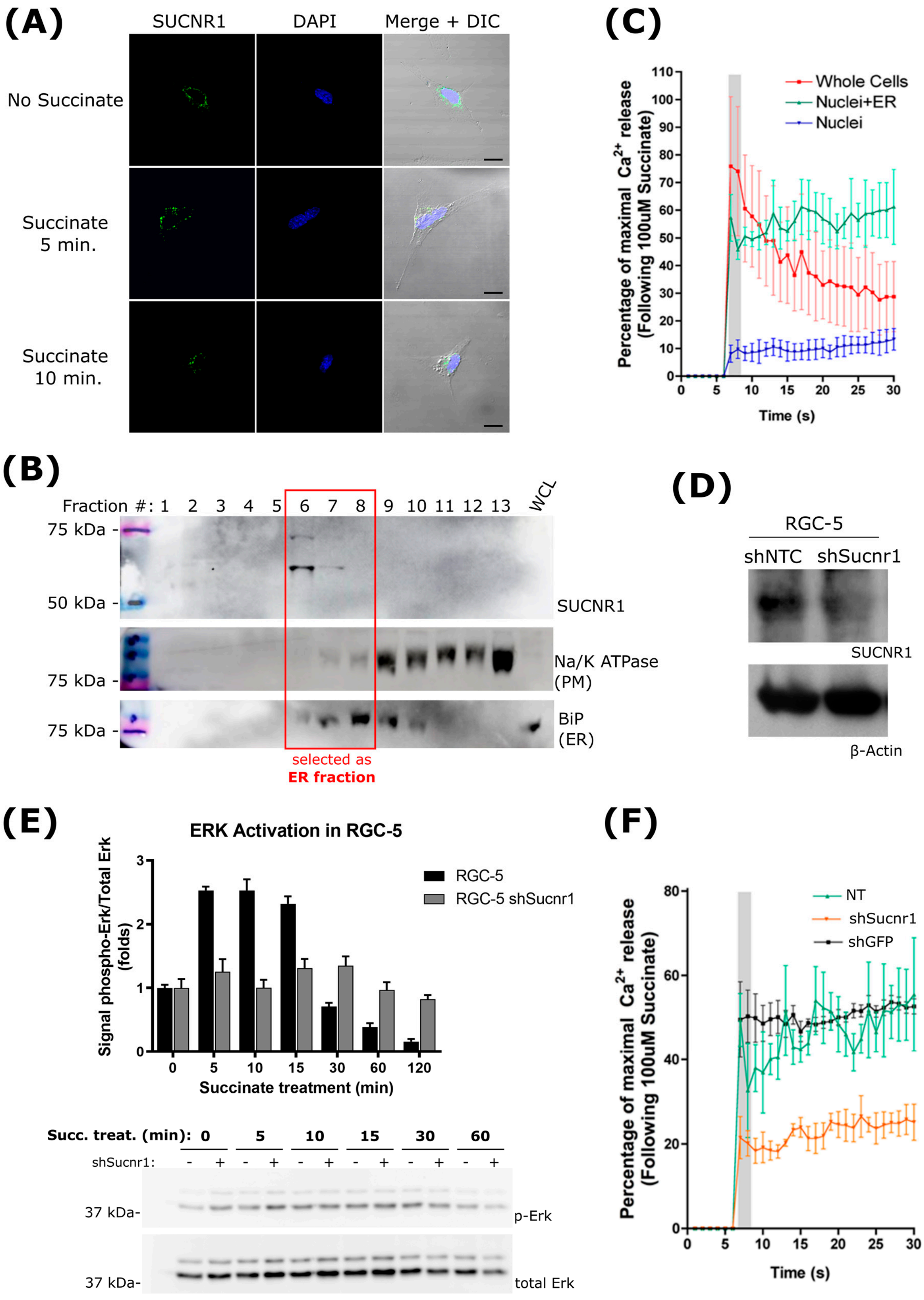
(A)
SUCNR1
DAPI
Merge + DIC
No Succinate
Succinate 5 min.
Succinate 10 min.
(C)
Percentage of maximal Ca2+ release (Following 100uM Succinate)
Whole Cells
Nuclei+ER
Nuclei
Time (s)
(B)
Fraction #: 1 2 3 4 5 6 7 8 9 10 11 12 13 WCL
75 kDa
50 kDa
SUCNR1
Na/K ATPase (PM)
BiP (ER)
selected as
ER fraction
(D)
RGC-5
shNTC
shSucnr1
SUCNR1
β-Actin
(E)
ERK Activation in RGC-5
Signal phospho-Erk/Total Erk (folds)
RGC-5
RGC-5 shSucnr1
Succinate treatment (min)
Succ. treat. (min): 0 5 10 15 30 60
shSucnr1:
37 kDa-
p-Erk
total Erk
(F)
Percentage of maximal Ca2+ release (Following 100uM Succinate)
NT
shSucnr1
shGFP
Time (s)

***Supplementary Figure S1**: Downstream pathway of SUCNR1 stimulation in RGC*

(**A**) Immunohistochemistry of endogenous SUCNR1 in RGC-5 cells treated with 100 µM succinate for 5 or 10 minutes. SUCNR1 signal is shown in green, and cell nuclei, stained with DAPI, shown in blue. Scale bars: 20 µm. (**B**) Representative western-blot pictures of cell fractions purity. Each lane represents a sedimentation fraction. Na/K-ATPase and BiP immunoblots reveal the PM and ER-containing fractions, respectively. Fractions with the strongest BiP signal and least Na/K-ATPase signal were selected as ER fraction. (**C**) Related to **figure 1D**. FURA-2 fluorescence measurements upon succinate stimulation of RGC cell fractions shows calcium release from WC and Nucl+ER fractions but not from Nuclei only. (**D**) SUCNR1 and actin-b immunoblots of cell lysates from RGC-5 cells transfected with a non-targeting control (shNTC) or a SUCNR1-specific shRNA show that shSucnr1 expression efficiently decreases SUCNR1 level. (**E**) RGC cells treated with 100 µM succinate show a transient ERK activation by western blot, which is impaired when *Sucnr1* is knocked-down. For each time-point, quantification shows the signal ratio of phospho-ERK 1/2 to total ERK 1/2, relative to the initial time point. (**F**) Related to **figure 1F**. FURA-2 fluorescence measurements upon succinate stimulation of RGC cell Nuclei+ER fractions shows calcium release is impaired in cells carrying a shSucnr1 plasmid.

(A)

species	human N8 cons.	human N168 cons.	C-terminus	UniProt ID
Hs:	$_{5}$MAW**NAT**CKN$_{13}$	$_{163}$PVITD**NGT**T$_{171}$	$_{326}$ELLLSFREK$_{334}$	Q9BXA5
Mm:	$_{1}$MAQ**NLS**CEN$_{9}$	$_{162}$KEEGSNCID$_{170}$	$_{309}$FKSLTSFRT$_{317}$	Q99MT6
Rn:	$_{1}$MAQ**NLS**CEN$_{9}$	$_{162}$IEKGDSCVD$_{170}$	$_{309}$FKSLTSFRL$_{317}$	Q6IYF9
Ss:	$_{1}$MAS**NAT**CKN$_{9}$	$_{159}$PMIAV**NST**S$_{167}$	$_{310}$FKFLTSFRR$_{318}$	I3LMZ5
Gg:	$_{1}$MAV**NGT**DGC$_{9}$	$_{159}$VSPAN**NFT**C$_{167}$	$_{309}$LGRLKSTGK$_{317}$	A0A3Q2U480

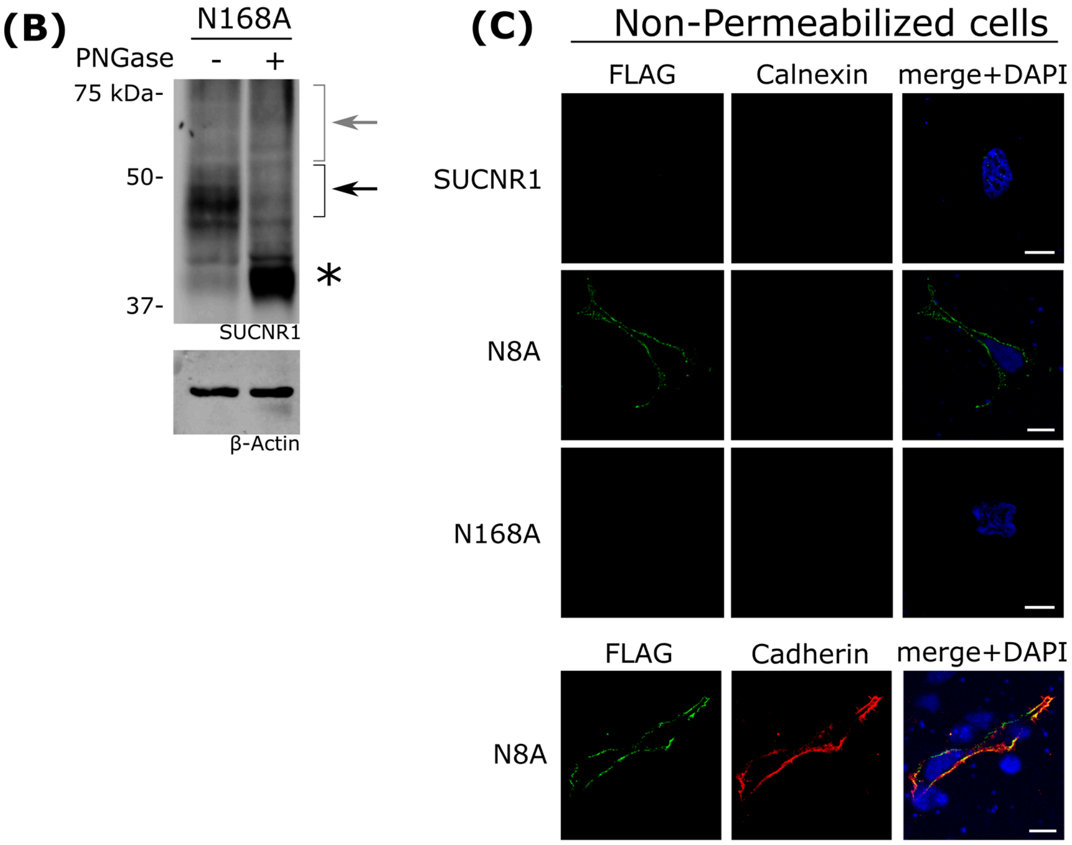

***Supplementary Figure S2**: SUCNR1 phylogenetic alignment and N-glycosylation mutant localization*

(**A**) Excerpts of the relevant signals from the multiple sequence alignment (MSA) of the Human, Mouse, Rat, Pig, and Chicken (*Hs*, *Mm*, *Rn*, *Ss*, and *Gg*, resp.) SUCNR1 protein sequences. Amino-acids (aa) numbering is shown below the first and last aa of each excerpt. Provided sequences are the N-terminal and ECL2 N-glycosylation sites (indicated in bold), showing the former is conserved but not the latter. Also shown are the C-terminal sequences showing that none contains the canonical KKXX sequence, and the Uniprot ID used for the MSA. (**B**) Related to **figure 2C**. Transfected HEK 293T whole cell lysates were treated with PNGaseF to fully deglycosylate proteins. After treatment, SUCNR1 migrates to its theoretical molecular weight of 37 KDa (*). Fully glycosylated and partially glycosylated receptors are indicated by grey and black arrows, resp. (**C**) Related to **figure 2D**. Cellular localization of FLAG-SUCNR1 by confocal microscopy in Non-Permeabilized cells. HEK 293T cells were transfected with FLAG-tagged wt-, N8A-, or N168A mutant SUCNR1 constructs and subjected to IF against FLAG tags (shown in green).

Calnexin was used as an ER marker, pan-cadherin was used as a Plasma-membrane marker (red), and nuclei were stained with DAPI (blue). Scale bars: 20 µm.

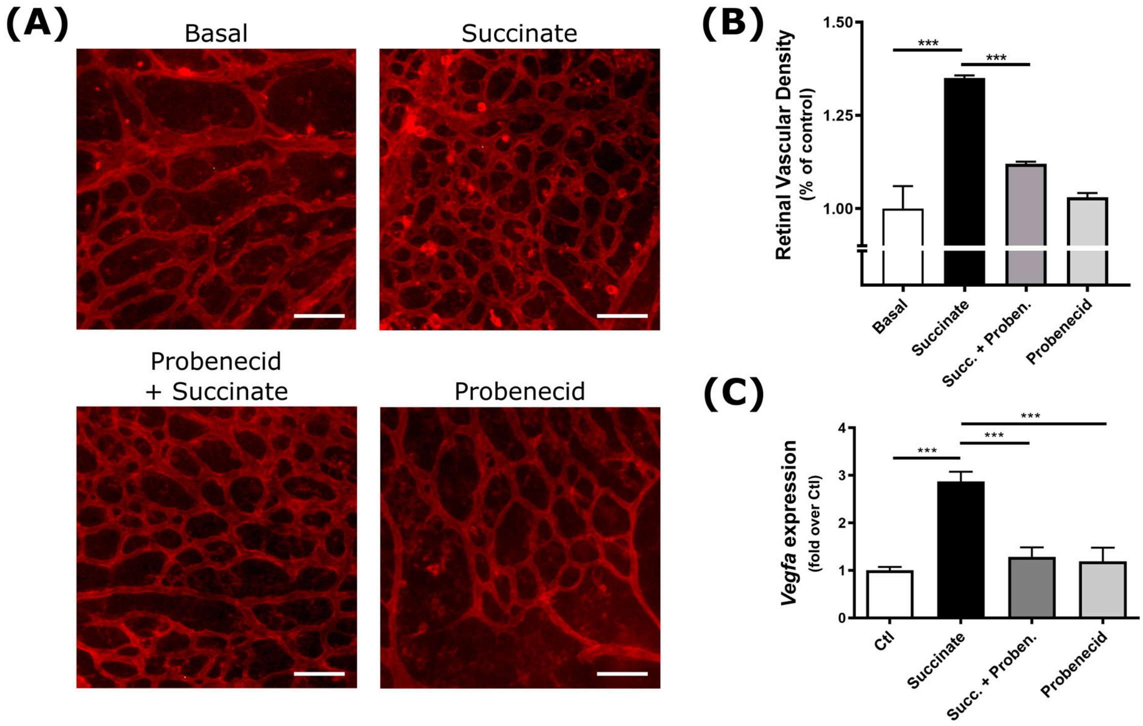

Supplementary Figure S3: *Probenecid impairs succinate-induced vascularization and Vegfa expression*

(**A**) Representative pictures of lectin-stained superficial retina vessels from P8 WT mice injected or not at P4 with succinate (100 µM final concentration), and with or without the organic anion transporters inhibitor probenecid (1mM final concentration). Scale bars: 100 µm. (**B**) Quantifications of superficial retina vascular density prepared as in (A). (**C**) In RGC-5 cells, *Vegfa* expression increases after a 4-hours 100 µM succinate treatment (black bar), co-administration of Probenecid abolishes this expression (dark grey). ***p<0.001 vs comparative value.

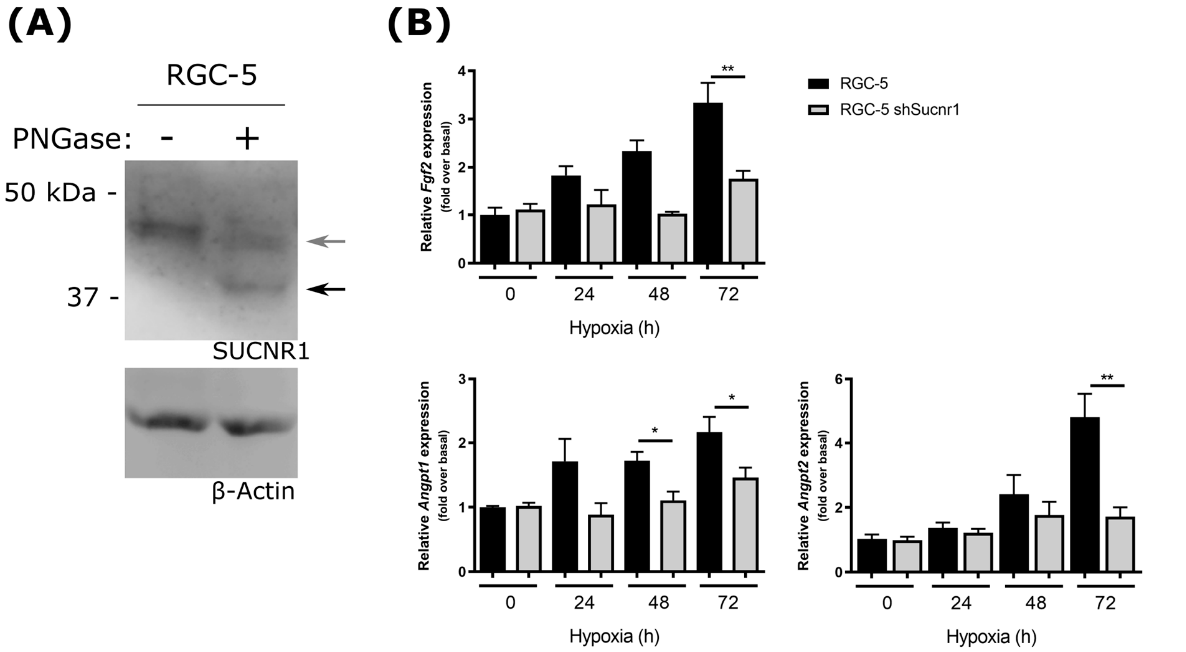

Supplementary Figure S4: *SUCNR1 deglycosylation in hypoxia affects downstream gene expression in RGC-5 cells*

(**A**) SUCNR1 is glycosylated in RGC. RGC-5 cell lysates were treated with PNGaseF, which led to a migration shift of SUCNR1 from its glycosylated form (grey arrow) to its theoretical molecular weight of 37 KDa (black arrow). (**B**) Gene expression in cells knocked-down for SUCNR1 during hypoxia. RGC-5 cells expressing an shRNA against SUCNR1 or not were cultured in hypoxic conditions for the indicated times before lysis and RT-qPCR quantification of *Fgf2*, *Angpt1*, and *Angpt2* expression. Black bars: shCTL ; Grey bars: shSucnr1. N=4; *p<0.05, **p<0.01 vs comparative value.

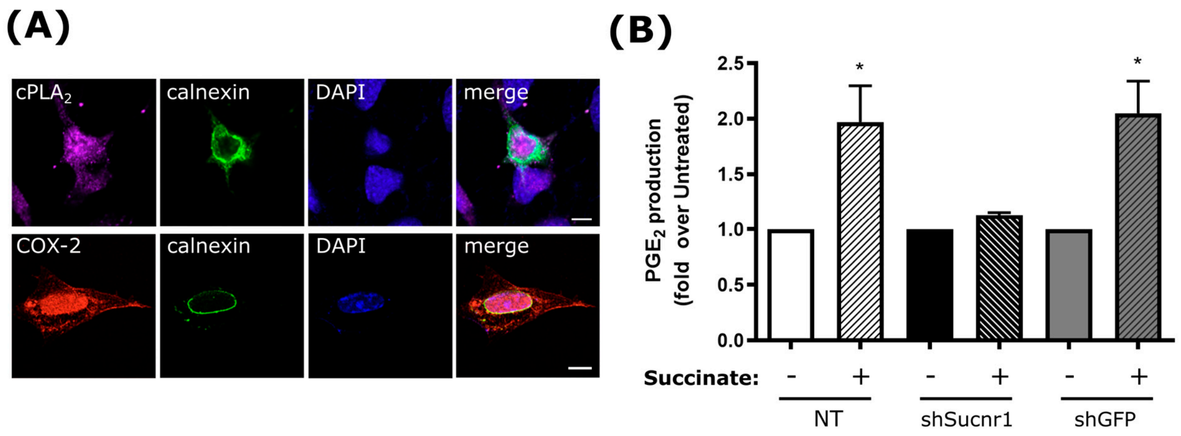

Supplementary Figure S5*: PGE production upon succinate treatment in RGC*

(**A**) In RGC-5 cells, COX-2 and $cPLA_2$ immunofluorescence show a signal colocalizing with both nuclei (DAPI staining, blue) and calnexin (IF, green). Scale bars: 20 µm. (**B**) ELISA quantification of PGE_2 in RGC-5 cells non-transfected (white bars), expressing an shRNA construct against SUCNR1 (black bars), or GFP (grey bars), and treated (striped bars) or not (plain bars) with Succinate; NT: non-transfected. N=4; *p<0.05 vs comparative value.
